# Are Ambient Ultrafine, Accumulation Mode, and Fine Particles Associated with Adverse Cardiac Responses in Patients Undergoing Cardiac Rehabilitation?

**DOI:** 10.1289/ehp.1104262

**Published:** 2012-04-27

**Authors:** David Q Rich, Wojciech Zareba, William Beckett, Philip K Hopke, David Oakes, Mark W Frampton, John Bisognano, David Chalupa, Jan Bausch, Karen O’Shea, Yungang Wang, Mark J Utell

**Affiliations:** 1 Department of Community and Preventive Medicine, and; 2 Division of Cardiology, Department of Medicine, University of Rochester Medical Center, Rochester, New York, USA; 3 Department of Medicine, Mount Auburn Hospital, Cambridge, Massachusetts, USA; 4 Department of Chemical and Biomolecular Engineering, Clarkson University, Potsdam, New York, USA; 5 Department of Biostatistics and Computational Biology, and; 6 Division of Pulmonary and Critical Care Medicine, Department of Medicine, University of Rochester Medical Center, Rochester, New York, USA

**Keywords:** air pollution, cardiac rehabilitation, fibrinogen, heart rate variability, repolarization

## Abstract

Background: Mechanisms underlying previously reported air pollution and cardiovascular (CV) morbidity associations remain poorly understood.

Objectives: We examined associations between markers of pathways thought to underlie these air pollution and CV associations and ambient particle concentrations in postinfarction patients.

Methods: We studied 76 patients, from June 2006 to November 2009, who participated in a 10-week cardiac rehabilitation program following a recent (within 3 months) myocardial infarction or unstable angina. Ambient ultrafine particle (UFP; 10–100 nm), accumulation mode particle (AMP; 100–500 nm), and fine particle concentrations (PM_2.5_; ≤ 2.5 μm in aerodynamic diameter) were monitored continuously. Continuous Holter electrocardiogram (ECG) recordings were made before and during supervised, graded, twice weekly, exercise sessions. A venous blood sample was collected and blood pressure was measured before sessions.

Results: Using mixed effects models, we observed adverse changes in rMSSD [square root of the mean of the sum of the squared differences between adjacent normal-to-normal (NN) intervals], SDNN (standard deviation of all NN beat intervals), TpTe (time from peak to end of T-wave), heart rate turbulence, systolic and diastolic blood pressures, C-reactive protein, and fibrinogen associated with interquartile range increases in UFP, AMP, and PM_2.5_ at 1 or more lag times within the previous 5 days. Exposures were not associated with MeanNN, heart-rate–corrected QT interval duration (QTc), deceleration capacity, and white blood cell count was not associated with UFP, AMP, and PM_2.5_ at any lag time.

Conclusions: In cardiac rehabilitation patients, particles were associated with subclinical decreases in parasympathetic modulation, prolongation of late repolarization duration, increased blood pressure, and systemic inflammation. It is possible that such changes could increase the risk of CV events in this susceptible population.

Multiple, interacting pathways (e.g. autonomic dysfunction, coagulation, inflammation, vascular dysfunction) have been investigated to explain previous findings of acute cardiovascular (CV) events following short-term increases in particulate pollution (Brook et al. 2004, 2010; Rich et al. 2010). Air pollution–mediated changes in biomarkers of these pathways (e.g. heart rate variability, fibrinogen, C-reactive protein [CRP]) have been observed in both elderly and healthy young subjects [Brook et al. 2010; Schneider et al. 2010; U.S. Environmental Protection Agency (EPA) 2009]. Although these PM-associated biomarker changes in healthy populations have generally been small and considered subclinical, they each suggest pathophysiologic mechanisms by which PM may acutely impact CV health. Further, in more health-compromised populations, such as cardiac rehabilitation patients, similarly sized effects may be clinically significant precursors to more severe CV events. Previous studies of patients with recent cardiac events have reported acute (within a few days), adverse changes in blood pressure, ST-segment depression, interleukin 6, and fibrinogen associated with ambient particle concentrations (Chuang et al. 2008; Rückerl 2007a; Zanobetti et al. 2004).

Ultrafine particles (UFP < 100 nm in diameter) have been associated with these and similar CV end points (Rückerl et al. 2007b; Schneider et al. 2010; Weichenthal et al. 2011). UFP may be particularly important with regard to CV effects because of their potential to enter the lung interstitium and vascular space directly and to evade clearance mechanisms. Previous studies have also reported more frequent or larger sized CV responses to accumulation model particles (AMP with diameters between 100 and 1,000 nm) than UFP and particles with an aerodynamic diameter ≤ 2.5 µm (PM_2.5_) (Anderson et al. 2008; Pekkanen et al. 2002).

To examine whether PM_2.5_, AMP, and UFP impact markers of pathways thought to underlie previous reports of cardiorespiratory mortality and morbidity, we conducted a panel study of cardiac rehabilitation patients who had a recent coronary event (myocardial infarction or unstable angina). We hypothesized that increases in ambient UFP, AMP, and PM_2.5_ concentrations in the previous few hours and days would result in decreased heart rate variability, impaired (decreased) baroreflex sensitivity, delayed repolarization, increased systemic inflammation, and increased systolic and diastolic blood pressure.

## Materials and Methods

*Study population.* We recruited 76 participants who were referred by their cardiologist to the University of Rochester Cardiac Rehabilitation Center (CR Center) after having a recent coronary event (MI or unstable angina). We excluded participants with cardiomyopathy in the absence of coronary disease, coronary bypass grafting within the last 3 months, type 1 diabetes, chronic atrial fibrillation, anemia, left bundle branch block, presence of a prosthetic heart valve or pacemaker, regular use of amiodarone, and active smokers or nonsmokers living with an active smoker. Participants lived within 19 km of the particle monitoring site at the CR Center (median of 9 km) and within 21 km of the New York State Department of Environmental Conservation (NYS DEC) particle monitoring site (median of 9 km).

*Study protocol.* Each participant underwent a 10-week supervised exercise program (≤ 20 exercise sessions) at the CR Center from June 2006 to November 2009. At each visit, participants were in the CR Center for 30–60 min before exercising and then seated for ≥ 5 min for blood pressure and electrocardiogram (ECG) recording. They warmed up for 2–5 min, which included gentle stretching, and then exercised for 30–45 min using a bicycle, treadmill, or rowing machine. After a “cool down” period, the participants rested for 10 min. Exercise modality was determined by the patients as part of their clinical rehabilitation program and not as part of the study. The same modality was used throughout the 10-week study period. The 76 participants and the data that were collected at their 1,489 participant visits were used in all analyses.

At each visit, participants underwent 3-lead (modified V2, V5, and AVF) Holter ECG recordings (Burdick Altair-Disc holter recorder; Cardiac Science, Bothell, WA), which were analyzed using the Vision Premier Burdick Holter System (Cardiac Science) and custom-made programs at the University of Rochester Medical Center, which have been described previously in our air pollution studies (Bauer et al. 2008; Cygankiewicz et al. 2008). All study Holters were annotated first automatically by the commercial Holter scanning algorithm (Vision Premier Burdick Holter System) and then annotated by a trained technician using standard procedures. RR intervals were exported to a custom made heart rate variability (HRV) program that produced a set of HRV parameters. Heart rate turbulence (HRT) and deceleration capacity (DC) were analyzed using programs adopted from Bauer et al. (2006) and from Schmidt et al. (1999). During both the preexercise resting period (~ 5 min seated) and for the entire recording (whole session was ~ 1–3 hr), we measured time domain HRV parameters including the mean normal-to-normal (NN) interval time between successive NN beats (MeanNN), the standard deviation of all NN beat intervals (SDNN), and the square root of the mean of the sum of squared differences between adjacent NN intervals (rMSSD). Short-term, preexercise, resting recordings provided information regarding HRV parameters unaffected by sympathetic stimuli during exercise, whereas the whole session recording (including the exercise session) reflected the overall behavior of heart rate and autonomic responses to daily conditions including the exercise. Based in part on Bigger et al. (1992), filtering criteria eliminated two RR intervals after premature ventricular or atrial beats. We did not apply preprocessing filtering to eliminate extreme values. We examined 5-min segments during the resting period to standardize conditions for all HRV and repolarization parameters, which required at least 200 beats for HRV analyses. As a postprocessing approach, we evaluated outliers and determined whether the values were valid or not based on intralab ranges developed during a prior study (Schneider et al. 2010).

Across the whole session, we measured HRT and DC. HRT, a measure of baroreflex sensitivity (Bauer et al. 2008; Cygankiewicz et al. 2008) is characterized by a brief acceleration and subsequent deceleration of heart rate following a spontaneous premature ventricular contraction. HRT is described by two parameters: turbulence onset and turbulence slope, and is associated with increased risk of cardiac death (Bauer et al. 2008). We focused on turbulence slope because previously this parameter was found to be more robust than turbulence onset in identifying participants with increased risk of cardiac events (Cygankiewicz et al. 2008). Because only 657 of the 1,489 recordings (from 72 of the 76 participants) had at least one premature ventricular beat (a mean of 126 ventricular ectopic beats per recording), we performed HRT analyses on a subset of recordings. Otherwise, all other outcomes were measured in all recordings. DC is an additional measure of heart rate dynamics, which reflects the variability in heart rate during periods when the heart is slowing down, complementing information based on the other HRV and HRT parameters (Bauer et al. 2008). Repolarization duration was analyzed using the QT interval duration, which was measured manually (i.e. technician evaluated 3 consecutive beats within each prespecified 2-min period from the beginning of the resting ECG in lead II, taking the average QT for each time-point), and corrected for heart rate (QTc) using Bazett’s formula (Bazett 1920). In addition, we also measured the Tpeak–Tend (TpTe), a measure of late repolarization duration, which may reflect heterogeneity in repolarization (Szydlo et al. 2010).

Blood pressure measurements were collected at each visit, and an atraumatically drawn venous blood sample was collected once weekly while the participant was resting and sitting before exercise. Blood pressure was measured by auscultation following a 5-min resting period with the arm supported at heart level. The blood pressure was measured three times as part of the research protocol, with an average used in the statistical analyses. Complete blood count, fibrinogen, and high sensitivity CRP analyses were measured in the Strong Memorial Hospital Clinical Laboratories (University of Rochester Medical Center, Rochester, NY). The study was approved by the Research Subjects Review Board of the University of Rochester, and informed written consent was obtained from all participants.

*Air pollution and weather measurements.* Particle size distributions for ultrafine particles (UFP; 10–100 nm diameter) and for accumulation mode particles (AMP; 100–500 nm diameter) were measured using a wide range particle spectrometer (model 1000XP; MSP Corporation, Shoreview, MN) at the CR Center from June 2006 to November 2009. The measured distributions have been summarized by Wang et al. (2010). Although the size range of the AMP is different from the standard 100–1,000-nm definition, the majority of particles in this size range are smaller in mass and closer to the 100-nm cut-off value. Therefore, the use of the 100–500-nm size range to define AMP rather than 100–1,000 nm should result in minimal difference in AMP concentration. This monitoring facility in Rochester is approximately 1,500 m from an interstate highway beltway. Concentrations of PM_2.5_ were measured using a tapered element oscillating microbalance (ThermoFisher, Franklin, MA) at the NYS DEC site in Rochester (~ 1.2 km from the CR Center). Hourly temperature, relative humidity, and barometric pressure were also measured at this same site.

*Statistical analysis.* We estimated the difference in each outcome (preexercise resting period: MeanNN, SDNN, rMSSD, QTc, and TpTe; whole session: MeanNN, SDNN, rMSSD, HRT, and DC; preexercise measurement: CRP, fibrinogen, white blood cell count, diastolic blood pressure, and systolic blood pressure) associated with each interquartile range (IQR) increase in pollutant concentration (UFP, AMP, PM_2.5_). Data were analyzed using mixed models, with the participants entered as random effects (version 9.2; PROC MIXED; SAS Institute Inc., Cary, NC). All analyses controlled for visit number, calendar time since the beginning of the study for each participant, month of year, and hour of day. Before estimating effects of the pollutants, we performed some initial analyses to select the appropriate correlation structure. For most outcomes, the compound symmetry covariance structure outperformed other structures examined (autoregressive and spatial power) according to the AIC criterion. Thus this structure was used in all subsequent analyses. Before assessing the effects of the particulate pollutants of interest, we examined other possible confounders including temperature, barometric pressure, relative humidity, sulfur dioxide, carbon monoxide, and ozone. To ensure comparability of analyses for the different outcomes, the same statistical model was used for each. Because only temperature showed effects that were frequently statistically significant, strongest at lag 0, this variable was included in all analyses.

Before the analysis, we log-transformed UFP and AMP to reduce skewness. We estimated changes in each outcome associated with each of the pollutant measures in separate analyses, using pollutants averaged over the 24-hr period before the visit as well as a shorter lag period (lag hr 0–5) and longer lag periods (lag hr 24–47, 48–71, 72–05, 96–119). We present the change in each outcome [and its 95% confidence interval (CI)] associated with an IQR increase in pollutant concentration during the specified lag period.

To examine whether a change in an outcome (e.g., increased TpTe) associated with one pollutant (e.g., AMP lagged 24–47) was independent of a second pollutant (UFP or PM_2.5_) at the same lag time, we used the same model described above (same covariates and correlation structure as single-pollutant model) including both pollutants at the lag time of interest (e.g., AMP and UFP counts lagged 24–47 hr). We then compared the parameter estimates from the single- and two-pollutant models.

We examined residual plots as a check on model assumptions. Where indicated, analyses were repeated after log-transformation of the relevant outcome variables. Statistical significance was defined as *p* < 0.05.

## Results

Of the 76 participants, 83% (*n* = 63) completed all 20 visits, with 8% (*n* = 6) completing < 10. Participants were generally, white, male, overweight, and former smokers (Table 1). Seventeen percent were < 50 years of age, and 21% were at least 70 years of age. Most had a history of MI, a prior stent, and a previous diagnosis of hypertension. Most were taking beta blockers, ACE inhibitors, and statins. Mean and SD levels of each outcome taken at the beginning of the first cardiac rehabilitation session are provided in Supplemental Material, Table S1 (http://dx.doi.org/10.1289/ehp.1104262). These mean baseline systolic and diastolic blood pressures were consistent with the findings of Zanobetti et al. (2004) in their study of patients in a cardiac rehabilitation program. The baseline inflammatory markers were within predicted normal ranges, and the baseline HRV parameters, although from only 1- to 3-hr recordings during exercise sessions, are within expected ranges.

**Table 1:** Characteristics of study population at baseline (n = 76).

Characteristic	n (%)
Age (years)	
< 50	13 (17)
50–59	21 (28)
60–69	26 (34)
70–79	14 (18)
≥ 80	2 (3)
Male	51 (67)
White	68 (88)
Body mass index (kg/m2)	
18.5 to < 25	10 (13)
25 to < 30	31 (41)
30 to < 35	22 (29)
≥ 35	13 (17)
Medical history	
Myocardial infarction	45 (59)
Coronary bypass surgery	4 (5)
Stent	65 (86)
Chronic obstructive pulmonary disease	13 (17)
Type 2 diabetes mellitus	17 (22)
Hypertension	45 (59)
Smoking	
Never	40 (53)
Former	36 (47)
Daily medication use at 1st visit	
Angiotensin receptor blockers	10 (13)
Beta blocker	66 (87)
Angiotensin-converting-enzyme inhibitor	50 (66)
Calcium channel blocker	7 (9)
Digitalis	1 (1)
Diuretic	20 (26)
Statin	73 (96)

Descriptive statistics for each pollutant and weather conditions are shown in Table 2. The UFP and AMP concentrations measured at the CR Center are similar to those seen across Rochester, New York, previously, and in a number of other U.S. locations (Wang et al. 2011). PM_2.5_ concentrations were similar to those in other northeastern U.S. cities (U.S. EPA 2009). AMP was moderately well correlated with both UFP (*r* = 0.51) and PM_2.5_ (*r* = 0.62), but UFP and PM_2.5_ were not (*r* = 0.11). UFP, AMP, and PM_2.5_ were less well correlated with temperature and relative humidity (*r's* ≤ 0.19). The IQR for the 6-hr mean UFP count (2,885 particles/cm^3^), AMP count (897 particles/cm^3^), and PM_2.5_ concentration (7.2 µg/m^3^) were used to scale all lag hr 0–5 effect estimates and CIs. The IQRs for the 24-hr mean UFP count (2,680 particles/cm^3^), AMP count (838 particles/cm^3^), and PM_2.5_ concentration (6.5 µg/m^3^) were used to scale all lag hr 0–23, 24–47, 48–71, 72–95, and 96–119 effect estimates and confidence intervals.

**Table 2:** Descriptive statistics of daily air pollution concentrations and weather characteristics during the study period (26 June 2006 to 25 November 2009).

Pollutant/weather characteristic	na	Mean ± SD	Minimum	25th percentile	Median	75th percentile	Maximum
Temperature (°C)	1,249	11.3 ± 10.1	–13.2	3.1	12.4	20.2	31.1
Relative humidity (%)	1,248	64.8 ± 13.4	10.2	56.8	65.7	73.6	95.3
Barometric pressure (inches Hg)	1,249	29.42 ± 0.26	27.45	29.26	29.42	29.59	30.12
Carbon monoxide (ppm)	1,187	0.411 ± 0.150	0.0083	0.312	0.392	0.492	1.046
PM2.5 (µg/m3)	1,135	8.67 ± 6.06	0.00	4.30	7.32	11.13	42.85
Sulfur dioxide (ppm)	1,123	0.0032 ± 0.0023	0.0000	0.0017	0.0026	0.0041	0.0259
Ozone (ppm)	1,222	0.0253 ± 0.0104	0.0008	0.0175	0.0240	0.0319	0.0648
UFP (10–100 nm; particles/cm3)	1,237	4,049 ± 2,168	328	2,518	3,623	5,166	16,767
AMP (100–500 nm; particles/cm3)	1,237	1,041 ± 783	20	505	858	1,371	6,314
a1,249 possible days of measurement.							

**Figure 1:**
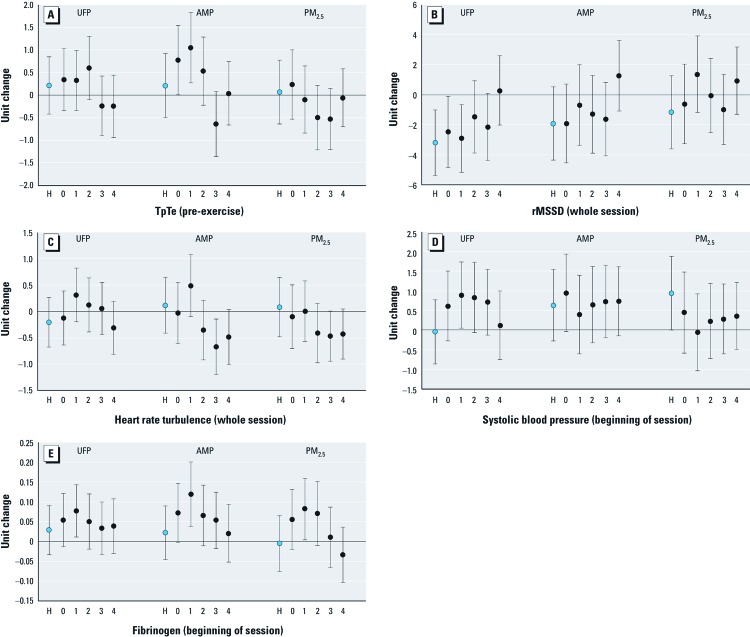
Unit change in TpTe measured at the beginning of the exercise session (*A*), rMSSD (*B*), and HRT measured across the whole session (*C*), and systolic blood pressure (*D*) and fibrinogen measured at the beginning of the exercise session (*E*), each with its 95% CI, associated with each IQR increase in UFP (10–100 nm), AMP (100–500 nm), and PM_2.5_ concentration, by lag hours. H, 6-hr mean, which is indicated by the blue circle (lag hr 0–5); 0, lag 0 (lag hr 0–23); 1, lag 1 (lag hr 24–47); 2, lag 2 (lag hr 48–71); 3, lag 3 (lag hr 72–95); 4, lag 4 (lag hr 96–119).

In Table 3, we present the changes in each outcome associated with IQR increases in UFP, AMP, and PM_2.5_ concentration in the previous five 24-hr lag periods, as well as in the previous 6 hr. There was no clear pattern of response to any pollutant for MeanNN or SDNN in the preexercise resting period, although we did observe a significant increase of 2.67 msec (95% CI: 0.88, 4.46) in SDNN associated with each IQR increase in PM_2.5_ 72–95 hr before the clinic visit. However, IQR increases in AMP in both the previous 6 and 24 hr were associated with significant decreases of 3.6 ms (95% CI: –6.39, –0.91) and 4.33 msec (95% CI: –7.27, –1.38) in rMSSD, respectively. Although not statistically significant, decreases in rMSSD were also associated with UFP and PM_2.5_ at the same lag times (Table 3). Although we observed no pattern of QTc duration response to any pollutant, each IQR increase in AMP was associated with 0.78 msec (95% CI: 0.02, 1.53) and 1.05 msec (95% CI: 0.28, 1.82) increases in TpTe in the previous 24 hr and 24–47 hr, respectively, before the clinic session (Table 3; Figure 1A).

**Table 3:** Change in each outcome, measured in the preexercise resting period, associated with each IQR increase in UFP, AMP, and PM_2.5_, by lag hour and time when outcome measurement was made.

Outcome and lag hr	UFP^a^		AMP^b^		PM_2.5_^c^	
	Change in outcome (95% CI)		Change in outcome (95% CI)		Change in outcome (95%CI)	
MeanNN (msec)									
0–5		–3.49	(–9.20, 2.23)		–1.21	(–7.61, 5.19)		–2.78	(–9.27, 3.70)
0–23		–2.53	(–8.76, 3.69)		–1.56	(–8.44, 5.33)		–0.21	(–7.41, 6.99)
24–47		–3.29	(–9.17, 2.59)		1.92	(–5.16, 9.00)		–0.08	(–7.05, 6.89)
48–71		3.40	(–2.89, 9.68)		3.37	(–3.47, 10.22)		0.52	(–6.19, 7.23)
72–95		3.71	(–2.14, 9.55)		1.75	(–4.64, 8.13)		–0.54	(–6.78, 5.70)
96–119		–3.45	(–9.56, 2.66)		–3.26	(–9.46, 2.95)		0.79	(–5.19, 6.77)
SDNN (msec)									
0–5		–1.07	(–2.72, 0.58)		–0.66	(–2.50, 1.19)		–1.37	(–3.25, 0.51)
0–23		–0.68	(–2.47, 1.12)		–0.68	(–2.66, 1.30)		0.12	(–1.96, 2.19)
24–47		0.05	(–1.65, 1.74)		0.58	(–1.46, 2.61)		1.61	(–0.39, 3.60)
48–71		–0.47	(–2.29, 1.35)		0.50	(–1.47, 2.47)		1.83#	(–0.09, 3.75)
72–95		–1.31	(–3.00, 0.38)		–0.44	(–2.29, 1.40)		2.67**	(0.88, 4.46)
96–119		–0.05	(–1.80, 1.71)		–0.01	(–1.79, 1.77)		0.68	(–1.03, 2.40)
rMSSD (msec)									
0–5		–2.31#	(–4.78, 0.16)		–3.65**	(–6.39, –0.91)		–2.81#	(–5.67, 0.06)
0–23		–2.45#	(–5.13, 0.24)		–4.33**	(–7.27, –1.38)		–1.53	(–4.67, 1.61)
24–47		–2.01	(–4.53, 0.52)		0.40	(–2.63, 3.43)		1.69	(–1.34, 4.73)
48–71		–0.91	(–3.64, 1.82)		–0.45	(–3.39, 2.49)		0.73	(–2.19, 3.66)
72–95		–1.74	(–4.29, 0.81)		–2.19	(–4.96, 0.57)		–0.22	(–2.95, 2.51)
96–119		–0.59	(–3.26, 2.09)		–1.87	(–4.58, 0.83)		–0.05	(–2.65, 2.55)
QTc (msec)									
0–5		0.43	(–1.03, 1.88)		0.17	(–1.44, 1.78)		–0.27	(–1.97, 1.42)
0–23		1.14	(–0.43, 2.71)		0.83	(–0.91, 2.57)		–0.13	(–1.98, 1.72)
24–47		0.17	(–1.34, 1.67)		–0.05	(–1.84, 1.74)		–0.93	(–2.72, 0.86)
48–71		0.19	(–1.40, 1.77)		0.85	(–0.87, 2.58)		0.23	(–1.49, 1.95)
72–95		–1.09	(–2.59, 0.42)		–0.68	(–2.32, 0.97)		–0.04	(–1.64, 1.57)
96–119		0.53	(–1.05, 2.10)		1.02	(–0.58, 2.62)		0.56	(–0.97, 2.09)
TpTe (msec)									
0–5		0.21	(–0.41, 0.84)		0.21	(–0.49, 0.91)		0.07	(–0.63, 0.76)
0–23		0.34	(–0.33, 1.02)		0.78*	(0.02, 1.53)		0.24	(–0.52, 0.99)
24–47		0.33	(–0.32, 0.98)		1.05**	(0.28, 1.82)		–0.10	(–0.83, 0.63)
48–71		0.60#	(–0.09, 1.29)		0.53	(–0.22, 1.28)		–0.50	(–1.20, 0.20)
72–95		–0.24	(–0.89, 0.41)		–0.64#	(–1.35, 0.07)		–0.53	(–1.19, 0.14)
96–119		–0.25	(–0.92, 0.43)		0.04	(–0.65, 0.72)		–0.06	(–0.69, 0.57)
aIQR increases of 2,885 particles/cm3 (6-hr mean) and 2,680 particles/cm3 (24-hr mean). bIQR increases of 897 particles/cm3 (6-hr mean) and 838 particles/cm3 (24-hr mean). cIQR increases of 7.2 µg/m3 (6-hr mean) and 6.5 µg/m3 (24-hr mean). *p < 0.05. **p < 0.01. #p < 0.10.									

We found no clear pattern of response of MeanNN or DC to any pollutant across the whole session (Table 4). However, each IQR increase in UFP 24–47 hr before the clinic session was associated with a 2.19 msec (95% CI: –4.16, –0.22) decrease in SDNN. We also found that rMSSD decreased significantly with the same IQR increase in UFP within the previous 48 hr (lag hr 0–5, 0–23, and 24–47), with the largest change (–3.19 msec; 95% CI: –5.32, –1.05) observed with the UFP count in the 6 hr before the exercise session (Table 4, Figure 1B). Each IQR increase in AMP 72–95 hr before the clinic session was also associated with a significant 0.67 msec/RR (95% CI: –1.18, –0.15) reduction in HRT. There were similar, albeit nonsignificant, reductions in HRT associated with increases in PM_2.5_ concentration during the same time period, but not in UFP (Table 4, Figure 1C).

**Table 4:** Change in each outcome, measured across the whole session, associated with each IQR increase in UFP, AMP, and PM_2.5_, by lag hour and time when outcome measurement was made.

UFP^a^		AMP^b^		PM_2.5_^c^	
Outcome and lag hr	Change in outcome (95% CI)		Change in outcome (95% CI)		Change in outcome (95% CI)	
MeanNN (msec)									
0–5		–1.42	(–5.67, 2.83)		–0.17	(–4.92, 4.59)		0.29	(–4.47, 5.05)
0–23		–1.69	(–6.33, 2.94)		–0.50	(–5.62, 4.62)		0.73	(–4.52, 5.97)
24–47		–2.88	(–7.27, 1.51)		–0.33	(–5.56, 4.90)		–0.47	(–5.48, 4.55)
48–71		0.76	(–3.90, 5.42)		–1.36	(–6.43, 3.71)		–2.74	(–7.59, 2.10)
72–95		2.02	(–2.30, 6.35)		1.11	(–3.65, 5.87)		–0.55	(–5.07, 3.96)
96–119		0.38	(–4.12, 4.89)		–0.74	(–5.32, 3.84)		–1.37	(–5.69, 2.95)
SDNN (msec)									
0–5		–0.84	(–2.75, 1.06)		–1.29	(–3.43, 0.84)		–0.82	(–2.98, 1.33)
0–23		–1.90#	(–3.98, 0.18)		–1.42	(–3.72, 0.88)		0.08	(–2.30, 2.45)
24–47		–2.19*	(–4.16, –0.22)		–0.04	(–2.39, 2.31)		0.65	(–1.64, 2.93)
48–71		0.13	(–1.96, 2.22)		1.38	(–0.89, 3.65)		1.67	(–0.53, 3.88)
72–95		1.03	(–0.91, 2.98)		–0.09	(–2.23, 2.04)		0.12	(–1.94, 2.18)
96–119		1.09	(–0.93, 3.12)		–0.05	(–2.11, 2.00)		0.20	(–1.77, 2.18)
rMSSD (msec)									
0–5		–3.19**	(–5.32, –1.05)		–1.91	(–4.31, 0.49)		–1.14	(–3.53, 1.25)
0–23		–2.46*	(–4.79, –0.13)		–1.90	(–4.48, 0.68)		–0.62	(–3.24, 2.00)
24–47		–2.89*	(–5.10, –0.68)		–0.69	(–3.33, 1.95)		1.37	(–1.14, 3.89)
48–71		–1.47	(–3.83, 0.89)		–1.28	(–3.83, 1.27)		–0.06	(–2.49, 2.37)
72–95		–2.14#	(–4.33, 0.05)		–1.62	(–4.02, 0.79)		–0.98	(–3.28, 1.33)
96–119		0.27	(–2.00, 2.54)		1.27	(–1.03, 3.57)		0.93	(–1.27, 3.13)
HRT (msec/RR)d									
0–5		–0.20	(–0.67, 0.26)		0.12	(–0.40, 0.64)		0.08	(–0.47, 0.64)
0–23		–0.13	(–0.63, 0.38)		–0.03	(–0.60, 0.54)		–0.10	(–0.69, 0.50)
24–47		0.31	(–0.19, 0.81)		0.49#	(–0.09, 1.07)		0.01	(–0.56, 0.57)
48–71		0.13	(–0.38, 0.63)		–0.35	(–0.91, 0.20)		–0.41	(–0.96, 0.14)
72–95		0.06	(–0.43, 0.55)		–0.67*	(–1.18, –0.15)		–0.46#	(–0.93, 0.00)
96–119		–0.31	(–0.80, 0.18)		–0.48#	(–0.99, 0.03)		–0.42#	(–0.89, 0.04)
DC (msec)									
0–5		0.014	(–0.055, 0.083)		–0.046	(–0.123, 0.031)		–0.038	(–0.117, 0.042)
0–23		–0.002	(–0.078, 0.074)		–0.038	(–0.120, 0.044)		–0.025	(–0.112, 0.061)
24–47		0.009	(–0.062, 0.080)		–0.041	(–0.125, 0.044)		–0.025	(–0.108, 0.059)
48–71		–0.015	(–0.091, 0.062)		–0.068	(–0.150, 0.014)		–0.048	(–0.129, 0.032)
72–95		0.024	(–0.047, 0.094)		–0.027	(–0.104, 0.050)		–0.019	(–0.094, 0.057)
96–119		–0.008	(–0.081, 0.065)		–0.001	(–0.075, 0.072)		0.022	(–0.050, 0.094)
aIQR increases of 2,885 particles/cm3 (6-hr mean) and 2,680 particles/cm3 (24-hr mean). bIQR increases of 897 particles/cm3 (6-hr mean) and 838 particles/cm3 (24-hr mean). cIQR increases of 7.2 µg/m3 (6-hr mean) and 6.5 µg/m3 (24-hr mean). dBecause HRT is measured only when premature ventricular contractions (PVC) occur, these analyses include only those subject with ≥ 1 PVC. *p < 0.05. **p < 0.01. #p < 0.10.									

We observed increases in systolic blood pressure associated with each IQR increase in UFP, AMP, and PM_2.5_ at almost all lags, of which, the largest were significant 0.89 mmHg (95% CI: 0.06, 1.72) and 0.94 mmHg (95% CI: 0.02, 1.87) increases associated with IQR increases in UFP lagged 24–47 hr and PM_2.5_ lagged 0–5 hr, respectively (Table 5, Figure 1D). However, this pattern was not as clear with diastolic blood pressure, as each IQR increase in UFP lagged 96–119 hr was associated with significantly decreased diastolic blood pressure (Table 5).

**Table 5:** Change in each outcome, measured at the beginning of the exercise session, associated with each IQR increase in UFP, AMP, and PM_2.5_, by lag hour and time when outcome measurement was made.

UFP^a^		AMP^b^		PM_2.5_^c^	
Outcome and lag hr		Change in outcome (95% CI)		Change in outcome (95% CI)		Change in outcome (95% CI)	
DBP (mmHg)									
0–5		–0.14	(–0.62, 0.35)		0.21	(–0.33, 0.75)		0.00	(–0.55, 0.56)
0–23		0.18	(–0.35, 0.71)		0.29	(–0.29, 0.88)		–0.25	(–0.86, 0.36)
24–47		0.11	(–0.40, 0.61)		–0.09	(–0.69, 0.51)		–0.12	(–0.70, 0.46)
48–71		0.31	(–0.23, 0.84)		0.02	(–0.56, 0.59)		0.07	(–0.50, 0.63)
72–95		0.33	(–0.16, 0.83)		–0.33	(–0.87, 0.21)		–0.15	(–0.67, 0.37)
96–119		–0.53*	(–1.04, –0.01)		–0.30	(–0.82, 0.22)		0.28	(–0.22, 0.78)
SBP (mmHg)									
0–5		–0.04	(–0.84, 0.76)		0.63	(–0.27, 1.53)		0.94*	(0.02, 1.87)
0–23		0.61	(–0.26, 1.49)		0.95#	(–0.02, 1.92)		0.45	(–0.56, 1.47
24–47		0.89*	(0.06, 1.72)		0.40	(–0.60, 1.39)		–0.05	(–1.02, 0.92)
48–71		0.83#	(–0.05, 1.72)		0.65	(–0.31, 1.62)		0.23	(–0.71, 1.17)
72–95		0.72#	(–0.11, 1.54)		0.73	(–0.17, 1.64)		0.29	(–0.59, 1.16)
96–119		0.12	(–0.74, 0.98)		0.74#	(–0.13, 1.60)		0.36	(–0.47, 1.20)
WBC (× 109/L)									
0–5		0.064	(–0.039, 0.168)		–0.055	(–0.169, 0.058)		–0.072	(–0.182, 0.037)
0–23		0.040	(–0.072, 0.152)		–0.028	(–0.155, 0.098)		–0.047	(–0.168, 0.073)
24–47		0.031	(–0.079, 0.141)		–0.009	(–0.145, 0.128)		0.015	(–0.105, 0.136)
48–71		0.031	(–0.086, 0.148)		–0.073	(–0.203, 0.057)		–0.049	(–0.174, 0.077)
72–95		0.020	(–0.089, 0.130)		–0.031	(–0.149, 0.088)		–0.051	(–0.164, 0.062)
96–119		0.032	(–0.083, 0.148)		0.063	(–0.059, 0.185)		0.047	(–0.061, 0.154)
CRP (mg/L)									
0–5		0.012	(–0.043, 0.068)		0.046	(–0.015, 0.106)		0.012	(–0.052, 0.076)
0–23		0.039	(–0.021, 0.100)		0.063#	(–0.004, 0.131)		0.033	(–0.037, 0.102)
24–47		0.052#	(–0.007, 0.112)		0.063#	(–0.011, 0.137)		0.023	(–0.047, 0.094)
48–71		–0.014	(–0.077, 0.048)		–0.018	(–0.087, 0.051)		0.021	(–0.052, 0.095)
72–95		0.005	(–0.054, 0.064)		0.021	(–0.043, 0.084)		0.069*	(0.003, 0.135)
96–119		0.032	(–0.030, 0.094)		0.014	(–0.052, 0.079)		0.001	(–0.060, 0.061)
Fibrinogen (g/L)									
0–5		0.029	(–0.032, 0.090)		0.023	(–0.043, 0.089)		–0.004	(–0.074, 0.065)
0–23		0.054	(–0.012, 0.120)		0.072#	(–0.001, 0.146)		0.056	(–0.019, 0.130)
24–47		0.078*	(0.013, 0.143)		1.120**	(0.039, 0.201)		0.082*	(0.006, 0.159)
48–71		0.051	(–0.018, 0.120)		0.066#	(–0.009, 0.141)		0.071#	(–0.008, 0.150)
72–95		0.034	(–0.031, 0.099)		0.054	(–0.016, 0.124)		0.011	(–0.064, 0.086)
96–119		0.040	(–0.028, 0.107)		0.021	(–0.051, 0.093)		–0.033	(–0.102, 0.036)
aIQR increases of 2,885 particles/cm3 (6-hr mean) and 2,680 particles/cm3 (24-hr mean). bIQR increases of 897 particles/cm3 (6-hr mean) and 838 particles/cm3 (24-hr mean). cIQR increases of 7.2 µg/m3 (6-hr mean) and 6.5 µg/m3 (24-hr mean). *p < 0.05. **p < 0.01. #p < 0.10.									

White blood cell counts were not associated with any lagged pollutant concentration, but IQR increases in UFP, AMP, and PM_2.5_ concentrations were associated with increases in CRP and fibrinogen at most lags, although only a few associations were statistically significant (Table 5). Each IQR increase in PM_2.5_ was associated with a significant increase of 0.069 mg/L (95% CI: 0.003, 0.135) in CRP lagged 72–95 hr, whereas each IQR increase in AMP lagged 24–47 hr was associated with a significant 0.120 g/L (95% CI: 0.039, 0.201) increase in fibrinogen. Similar increases in fibrinogen were associated with IQR increases in UFP and PM_2.5_ concentrations at the same lag time (Table 5, Figure 1E).

Next we evaluated whether associations were robust to include a second pollutant in the model by comparing pollutant specific effect estimates from the single- and two-pollutant models [see Supplemental Material, Table S2 (http://dx.doi.org/10.1289/ehp.1104262)]. Our findings of increased TpTe associated with AMP lagged 24–47 hr, decreased HRT associated with increased AMP lagged 72–95 hr, and increased fibrinogen associated with increased AMP lagged 24–47 hr, all appeared independent of other pollutants at the same lag times, because there were only small changes in the AMP effect estimates when controlling for either UFP or PM_2.5_. In contrast, changes in TpTe, HRT, and fibrinogen associated with lagged UFP or PM_2.5_ were generally reduced when controlling for AMP, with the exception of the change in HRT associated with UFP, which was larger after adjusting for AMP. Similarly, the decreased rMSSD associated with UFP in the previous 5 hr and the increased SBP associated with increased PM_2.5_ lagged 0–5 hr both appeared independent of AMP, as the UFP and PM_2.5_ effect estimates were little changed from the single-pollutant model to the two-pollutant model including AMP (see Supplemental Material, Table S2).

## Discussion

In a panel of postinfarction patients who participated in a cardiac rehabilitation exercise program, we found significant adverse changes in SDNN, rMSSD, late repolarization duration (TpTe), HRT, systolic blood pressure, CRP, and fibrinogen associated with increases in at least one lagged UFP, AMP, and PM_2.5_ concentration. Although not all associations were statistically significant, we observed patterns of increased systolic blood pressure, TpTe, CRP, and fibrinogen associated with PM in the previous 3–4 days, decreased rMSSD and SDNN associated with increased PM in the previous few hours or days, and decreased HRT associated with increased PM during the previous 2–4 days. Associations were more common and parameter estimates generally larger for lagged AMP and UFP concentrations than for PM_2.5_ concentrations. Associations were independent of calendar time since inception of the study for each participant, calendar month, hour of the day, temperature, and duration of participation in the rehabilitation program. Further, the associations with AMP and UFP were similar after adjusting for PM_2.5_.

Heart rate variability, together with HRT and DC, represent a set of variables providing insight into autonomic regulation and baroreflex response of the CV system. Impaired autonomic regulation of the heart is observed in numerous conditions (e.g., disease processes such as ischemic heart disease, myocardial infarction, cardiomyopathies, diabetes, drug actions, and physiologic conditions such as stress, sleep, and exercise). Therefore, there are both short- and long-term changes in these autonomic nervous system measures. QTc and TpTe reflect repolarization, a critical mechanism of electrophysiology of cardiac cells that plays an important role in arrhythmogenesis and the risk of sudden death. Along with atrial and ventricular arrhythmias, QTc and TpTe reflect vulnerability of the myocardium and are highly variable. Acute increases in blood pressure increase myocardial work and oxygen consumption, and together with increased coagulation and inflammation, may worsen cardiac ischemia in postinfarction patients. Chronic, repeated exposures may contribute to long-term adverse effects, such as left ventricular hypertrophy, progression of coronary artery disease, and recurrent infarction.

UFP are an important component of combustion-related or secondary aerosol-related air pollution and have been associated with adverse vascular, inflammatory, and autonomic effects (Brook et al. 2010; U.S. EPA 2009). Current mass-based regulatory monitoring sites only measure ambient PM_2.5_ and PM_10_, and not UFP. UFP may be particularly important with regard to CV effects because their high specific surface area (Oberdörster et al. 1995), enhanced oxidant capacity (Brown et al. 2001; Li et al. 2003), and propensity to enter cells (Stearns et al. 1994), all of which may facilitate delivery of reactive chemical species to the pulmonary and systemic vasculature. The subset of AMP measured in this study (100–500 nm) includes the peak of the surface area distribution of the ambient aerosol. AMP have a lower deposition probability than do UFP, but regional deposition in the lung is primarily alveolar. Thus, material condensed onto the AMP surface can be effectively transported into the lungs and can provide a substantial dose of surface active materials to the alveolar space. The associations we observed are consistent with a greater CV response to ambient UFP and AMP than with PM_2.5_ mass concentrations. These observations are consistent with human controlled exposure studies suggesting vascular and thrombotic effects of UFP inhalation (Shah et al. 2008; Stewart et al. 2010).

We observed decreased rMSSD associated with UFP, AMP, and PM_2.5_ during both the preexercise period and across the whole session. During the preexercise period the autonomic nervous system is not fully physiologically activated (parasympathetic dominance), whereas the whole session recordings, which include daily activities and movements at the CR Center, reflect activation of sympathetic and baroreflex modulations in addition to parasympathetic modulation. Thus, whole session recordings reflect the natural interplay among these components of autonomic regulation. Our findings are consistent with a decrease in parasympathetic modulation of the heart. Associations were observed with exposures as recent as 6 hr before the exercise session, suggesting a rapid HRV response to UFP. Although previous studies are not entirely consistent, most have reported decreased HRV associated with increased PM in the previous few hours and days, with the strongest associations with the high frequency component of HRV (Brook et al. 2010). Because rMSSD is highly correlated with this high frequency component of HRV (Bigger et al. 1992), our findings of decreased rMSSD associated with increased UFP, AMP, and PM_2.5_ are consistent with these PM/high frequency associations.

We also assessed whether increased UFP, AMP, or PM_2.5_ concentrations were associated with adverse changes in HRT. We found a decreased HRT slope (i.e., impaired baroreflex sensitivity) associated with increased AMP concentration, which may lead to an increased risk of acute CV events including sudden cardiac death (Bauer et al. 2008; Cygankiewicz et al. 2008). HRT reflects baroreflex sensitivity, whereas DC is a nonspecific measure of overall HRV correlating with all indices of HRV. Therefore, it is not surprising that associations differed for HRT and DC because these outcomes do not represent the same mechanisms. DC is strongly vagally modulated, whereas HRT is driven by the baroreflex response.

In our analyses of repolarization, QTc evaluated at baseline was not associated with particulate air pollution at any lag. However, we did observe increased TpTe levels (considered as a measure of repolarization heterogeneity in the myocardium) associated with increased AMP in the previous 24 hr. Repolarization responses to ambient particulate air pollutant concentrations have been reported previously in humans (Brook et al. 2010; Henneberger 2005; Zanobetti et al. 2009; Zareba et al. 2009). Taken together, these findings suggest that cardiac rhythm is affected by ambient particulate air pollution, and specifically AMP.

Associations between increased systolic blood pressure and ambient particulate air pollution have not been consistently observed (Auchincloss et al. 2008; Brook et al. 2010; Ibald-Mulli 2004; Liu 2009). Our finding of increased systolic blood pressure associated with UFP and PM_2.5_ is consistent with that reported by Zanobetti et al. (2004) in a similar rehabilitation patient panel. However, the estimated effect size (1.37 mmHg systolic blood pressure associated with each 10.5-µg/m^3^ increase in PM_2.5_ concentration; scaled to the same IQR as in Zanobetti et al. (2004) and lag time of response that we observed (previous 6 hr) were smaller and earlier than reported in that study (2.8 mmHg systolic blood pressure associated with each 10.5-µg/m^3^ increase in mean PM_2.5_ concentration over the previous 5 days).

CRP and fibrinogen are “acute phase” proteins known to increase in the hours and days following an inflammatory stimulus and are associated with increased CV risk. Fibrinogen, in the presence of thrombin, forms fibrin, a key component of the blood clot. Short-term increases in these markers have been associated with increases in ambient particulate air pollution, especially in subjects with underlying CV risk factors (Brook et al. 2010; Rückerl et al. 2007a), but not in some controlled human exposure studies (Pietropaoli et al. 2004; Samet et al. 2009; U.S. EPA 2009). In patients with clinical coronary artery disease, however, we found significant increases in fibrinogen associated with all three particulate air pollutant size fractions in the previous 24–47 hr, providing evidence for particulate air pollution-mediated increases in systemic inflammation and coagulation in these susceptible subjects. Although the clinical significance of short-term increases in blood markers of inflammation has not been clearly established, it is possible that repeated increases in systemic inflammation in response to PM exposure may increase the risk for further events in patients with coronary artery disease.

Our study had a few limitations. First, although our inference was primarily based on the pattern of outcome responses to lagged ambient UFP, AMP, and PM_2.5_ concentrations, we did in fact estimate 270 pollutant–outcome associations. Thus, some of our 17 significant associations may be due to chance. Second, we used data from stations measuring PM_2.5_ and particle counts (UFP and AMP) and assigned concentrations from those sites to study participants regardless of how close they lived to the monitoring site, which may have resulted in some exposure error. However, this error is likely a combination of Berkson and classical error (Bateson et al. 2007; Zeger et al. 2000). Any classical error would result in a bias toward the null and underestimates of effect. Third, for our evaluation of the ECG parameters from the whole session, we allowed recordings to have a varying length, rather than a standard length. However, there was little variability in ECG recording length (95% of the recordings were 0.8–2.4 hr), and our effect estimates were little changed when excluding those participant visits with the shortest 5% (< 1 hr) and longest 5% (> 2 hr) of recording lengths (data not shown).

In this panel of patients with a recent acute coronary event who participated in a cardiac rehabilitation program, we found that increases in ambient UFP and AMP were associated with adverse changes in HRV, baroreflex sensitivity, blood pressure, cardiac repolarization, systemic inflammation, and blood coagulation within the previous few hours and days. The magnitudes of estimated effects for individual outcomes were small, but in combination may increase the risk of myocardial ischemia, contribute to parasympathetic withdrawal, and increase myocardial vulnerability to arrhythmias and postinfarction adverse remodeling that increase the risk of CV events in this susceptible population

## Supplemental Material

(156 KB) PDF
