# Correction: Are Ambient Ultrafine, Accumulation Mode, and Fine Particles Associated with Adverse Cardiac Responses in Patients Undergoing Cardiac Rehabilitation?

**DOI:** 10.1289/ehp.1104262-erratum

**Published:** 2012-09-01

On p. 1165 of “Are Ambient Ultrafine, Accumulation Mode, and Fine Particles Associated with Adverse Cardiac Responses in Patients Undergoing Cardiac Rehabilitation?” by Rich et al. [Environ Health Perspect 120:1162–1169 (2012)], four coefficients were incorrect. The corrected text is as follows:

AMP was moderately well correlated with both UFP (r = 0.51) and PM2.5 (r = 0.62), but UFP and PM2.5 were not (r = 0.11). UFP, AMP, and PM2.5 were less well correlated with temperature and relative humidity (r’s ≤ 0.19).

In addition, in Supplemental Material, Table S2 (http://dx.doi.org/10.1289/ehp.1104262), the 95% confidence interval was incorrect for the rMSSD (0–5 lag hr) for UFP in the two-pollutant model: “–6.47, 0.79” should have been “–6.47, –0.79.”

The authors apologize for the error.

These error have been corrected in the PDF version of this article and Supplemental Material.

