# Supplementary material for: Are Ambient Ultrafine, Accumulation Mode, and Fine Particles Associated with Adverse Cardiac Responses in Patients Undergoing Cardiac Rehabilitation?
Source: Environ Health Perspect. 2012 Apr 27;120(8):1162–9. doi: 10.1289/ehp.1104262 (PMC3440073; doi:10.1289/ehp.1104262)
Supplement: (156 KB) PDF [file ehp.1104262.s001.pdf]

SUPPLEMENTAL MATERIAL

Are Ambient Ultrafine, Accumulation Mode, and Fine Particles Associated With Adverse Cardiac Responses in Patients Undergoing Cardiac Rehabilitation?

David Q. Rich[1], Wojciech Zareba[2], William Beckett[3], Philip K. Hopke[4], David Oakes[5], Mark W. Frampton[6], John Bisognano[2], David Chalupa[6], Jan Bausch[5], Karen O'Shea[6], Yungang Wang[4], Mark J. Utell[6]

[1]Department of Community and Preventive Medicine, University of Rochester Medical Center, Rochester, NY

[2]Division of Cardiology, Department of Medicine, University of Rochester Medical Center, Rochester, NY

[3]Department of Medicine, Mount Auburn Hospital, Cambridge, MA

[4]Department of Chemical and Biomolecular Engineering, Clarkson University, Potsdam, NY

[5]Department of Biostatistics and Computational Biology, University of Rochester Medical Center, Rochester, NY

[6]Division of Pulmonary and Critical Care Medicine, Department of Medicine, University of Rochester Medical Center, Rochester, NY

Corresponding author: David Q. Rich, ScD
University of Rochester School of Medicine and Dentistry
Department of Community and Preventive Medicine
265 Crittenden Boulevard, CU 420644
Rochester, NY 14642
Phone: 585-276-4119
Fax: 585-424-1469
Email: david_rich@urmc.rochester.edu

Supplement Material, Table S1. Mean and standard deviation of each outcome, at the first cardiac rehabilitation visit.

**Outcome levels at baseline**	**Mean**	**Stand. Dev.**	*Minimum*	*Maximum*
**Pre-exercise resting period**				
MeanNN (ms)	944.77	141.32	*456.84*	*1433.04*
SDNN (ms)	55.60	29.36	*12.87*	*278.82*
rMSSD (ms)	60.52	44.45	*6.78*	*297.81*
QTc (ms)	419.81	32.56	*354.57*	*593.50*
TpTe (ms)	89.03	12.01	*55.89*	*149.25*
**Whole Session**				
MeanNN (ms)	733.32	110.40	*475.88*	*1102.68*
SDNN (ms)	132.07	43.32	*27.76*	*296.99*
RMSSD (ms)	77.70	38.15	*11.47*	*274.62*
Heart Rate Turbulence (ms/RR)	6.08	4.31	*0.00*	*19.50*
Deceleration Capacity (ms)	3.84	1.40	*0.00*	*9.79*
**Beginning of Session**				
Diastolic Blood Pressure (mmHg)	66.47	7.24	*50*	*96*
Systolic Blood Pressure (mmHg)	112.37	13.10	*80*	*179*
White Blood Cell Count ($x10^9$/L)	6.60	1.64	*3.10*	*13.80*
CRP (mg/L)	0.82	0.94	*0.01*	*7.50*
Fibrinogen (g/L)	3.58	0.87	*115*	*710*

Supplement Material, Table S2. Change (and 95% confidence interval) in each outcome associated with each interquartile range increase in UFP, AMP, and/or $PM_{2.5}$, for single and two pollutant models.

			SINGLE POLLUTANT MODELS*				TWO POLLUTANT MODEL			
Outcome	**Lag hours**	**Pollutant**	**N**	**Unit change**	**95% CI**	**p-value**	**n**	**Unit change**	**95% CI**	**p-value**
TpTe (ms)	24-47	AMP	1246	1.05	0.28,1.82	0.01	1246	1.23	0.29, 2.17	0.01
		UFP	1246	0.33	-0.32, 0.98	0.32		-0.26	-1.06, 0.53	0.51
		AMP	1246	1.05	0.28,1.82	0.01	1130	1.28	0.25, 2.31	0.01
		$PM_{2.5}$	1137	-0.10	-0.83, 0.63	0.79		-0.81	-1.75, 0.12	0.09
rMSSD (ms)	0-5	UFP	1346	-3.19	-5.32, -1.05	0.004	1346	-3.63	-6.47, -0.79	0.01
		AMP	1346	-1.91	-4.31, 0.49	0.12		-0.76	-2.42, 3.94	0.64
HRT (ms/RR)	72-95	AMP	504	-0.67	-1.18, -0.15	0.01	504	-1.05	-1.68, -0.42	0.001
		UFP	504	0.06	-0.43, 0.55	0.81		0.62	0.04, 1.21	0.04
		AMP	504	-0.67	-1.18, -0.15	0.01	467	-0.65	-1.39, 0.07	0.08
		$PM_{2.5}$	467	-0.46	-0.93, 0.00	0.05		-0.08	-0.71, 0.56	0.81
SBP (mmHg)	0-5	$PM_{2.5}$	1281	0.94	0.02, 1.87	0.05	1274	0.71	-0.52, 1.93	0.26
		AMP	1403	0.63	-0.27, 1.53	0.17		0.32	-0.94, 1.57	0.62
Fibrinogen (g/L)	24-47	AMP	641	0.120	0.039, 0.201	0.004	641	0.096	-0.003, 0.194	0.06
		UFP	641	0.078	0.013, 0.143	0.02		0.034	-0.045, 0.113	0.40
		AMP	641	0.120	0.039, 0.201	0.004	581	0.118	0.008, 0.228	0.04
		$PM_{2.5}$	584	0.082	0.006, 0.159	0.03		0.020	-0.077, 0.117	0.68

* Same results from Table 3-5. Presented again to more easily compare single and two pollutant model results

ERRATUM

NOTE: On p. 1165 of "Are Ambient Ultrafine, Accumulation Mode, and Fine Particles Associated with Adverse Cardiac Responses in Patients Undergoing Cardiac Rehabilitation?" by Rich et al. [Environ Health Perspect 120:1162–1169 (2012)], four coefficients were incorrect. The corrected text is as follows:

> AMP was moderately well correlated with both UFP (r = 0.51) and $PM_{2.5}$ (r = 0.62), but UFP and $PM_{2.5}$ were not (r = 0.11). UFP, AMP, and $PM_{2.5}$ were less well correlated with temperature and relative humidity (r's ≤ 0.19).

In addition, in Supplemental Material, Table S2 (http://dx.doi.org/10.1289/ehp.1104262), the 95% confidence interval was incorrect for the rMSSD (0–5 lag hr) for UFP in the two-pollutant model: "–6.47, 0.79" should have been "–6.47, –0.79."

The authors apologize for the error.

These error have been corrected in the PDF version of this article and Supplemental Material.
